# Effect of Birth Year on Birth Weight and Obesity in Adulthood: Comparison between Subjects Born Prior to and during the Great Depression in Iceland

**DOI:** 10.1371/journal.pone.0044551

**Published:** 2012-09-05

**Authors:** Cindy Mari Imai, Thorhallur Ingi Halldorsson, Ingibjorg Gunnarsdottir, Vilmundur Gudnason, Thor Aspelund, Gudmundur Jonsson, Bryndis Eva Birgisdottir, Inga Thorsdottir

**Affiliations:** 1 Unit for Nutrition Research, Landspitali University Hospital and Faculty of Food Science and Nutrition, School of Health Sciences, University of Iceland, Reykjavik, Iceland; 2 Icelandic Heart Assocation, Kopavogur, Iceland; 3 Faculty of Medicine, School of Health Sciences, University of Iceland, Reykjavik, Iceland; 4 Faculty of Humanities, Department of History and Philosophy, University of Iceland, Reykjavik, Iceland; John Hopkins Bloomberg School of Public Health, United States of America

## Abstract

**Background:**

Many epidemiological studies have linked small size at birth to adverse adult health outcomes but the relative influence of environmental exposures is less well established.

**Methods:**

The authors investigated the impact of prenatal environmental exposure by comparing 2750 participants born before (1925–1929) and during (1930–1934) the Great Depression in Reykjavik, Iceland. Calendar year served as proxy for environmental effects. Anthropometric measurements at birth and school-age (8–13 years) were collected from national registries. Participants were medically examined as adults (33–65 years).

**Results:**

Mean birth weight, adjusted for maternal age and parity, decreased by 97 g (95% confidence interval (CI): 39, 156) for men and 70 g (95% CI: 11, 129) for women from 1925 to 1934; growth at school-age was significantly reduced for participants growing during the Depression. As adults, women prenatally exposed to the Depression had higher body mass index (Δ0.6 kg/m^2^, 95% CI: 0.2, 1.1), higher fasting blood glucose levels (Δ0.16 mmol/L, 95% CI: 0.07, 0.23) and greater odds of being obese 1.43 (95% CI: 1.01, 2.02) compared to unexposed counterparts. Non-significant associations were observed in men.

**Conclusion:**

Reduction in birth weight due to rapid shifts in the economic environment appears to have a modest but significant association with later obesity for women while male offspring appear to be less affected by these conditions.

## Introduction

There is strong evidence suggesting risk of some metabolic disorders is set early in life and that these risks can be further heightened by factors in the external environment [1], [2]. An estimated 25–50% of normal variation in birth size is driven by genetics while environmental determinants contribute to the remaining influences [3], [4]. Exposure to an adverse intrauterine environment (i.e. undernutrition during pregnancy) has been proposed to lead not only to infants with low birth weight but also decreased muscle mass, with a disproportionately high fat to lean mass ratio [5].

The association between low birth weight and adverse adult health outcomes has been observed in well-nourished as well as famine exposed populations [6]–[9]. The Dutch famine studies showed that extreme fetal undernutrition is linked to glucose intolerance, cardiovascular disease, and mental health disorders in adulthood [10]–[12]. The first weeks of pregnancy (e.g. first trimester) appear to be the most vulnerable period and those exposed to famine during this time have consistently poorer health outcomes compared to unexposed individuals [12]. These findings have been reproduced in animal studies where mimicking prenatal malnutrition led to obesity and insulin resistance in offspring [13], [14]. However, it remains unclear from both animal and human studies the degree to which such perinatal conditions have a lasting effect.

The environment that humans live in can change swiftly and birth weight provides a snapshot of the intrauterine environment. Stressful events have been linked to adverse birth outcomes, particularly low birth weight. In the 1920s Iceland's economy was expanding until the onset of the Depression which affected the country drastically due to heavy reliance on exports [15]. The Great Depression hit the Western world in 1929. However, historical sources identify 1930 as the year the effects of the Depression reached Iceland forcing exports to lose up to 50% of their 1929 prices [15], [16] and declines in food supply data were reported for most years in the 1930s [17].

The rapid occurrence of the Depression in Iceland makes this time period relevant for investigating sudden environmental shifts and their impact on birth size under non-famine like conditions. The aim of this study was to identify the association between size at birth and later health prior to and after the onset of the Great Depression in a cohort of 2750 Icelanders born between 1925 and 1934. We hypothesized that those exposed to the Depression in utero would be of lower birth weight and have greater risk of disorders associated with smaller size at birth, specifically obesity and cardiometabolic risk factors.

## Materials and Methods

### Ethics Statement

The study protocol was granted approval by the Icelandic National Bioethics Committee and the Data Protection Commission and written informed consent was obtained from all study participants.

### Study Population

The source population for this study consists of 4601 subjects, aged 33–65 years, who were born in Reykjavik 1914–1935 and resided there when recruited into the longitudinal Reykjavik Study at the Icelandic Heart Association from 1967–1991. Methods regarding data collection have previously been described [18]. In brief, participants were randomly selected from the population register and invited to attend a clinical examination where anthropometrics, fasting blood samples, and medical history were collected by research personnel. Results on infant growth and later health have been previously published in this population [19]–[22], but environmental influences on birth weight have not been investigated.

To evaluate the impact of the Great Depression which began affecting Iceland in 1930, five year periods were combined (1925–1929 and 1930–1934) prior to and after the onset of the Depression as a proxy for environmental exposure. These two birth year groups, totaling 2750 participants or 60% of the source population, covers a span of ten years allowing the analysis of the immediate consequences of the Great Depression; subjects born greater than ten years apart will reflect other factors such as economic recovery, public health initiatives, medical advances as opposed to the specific influence of the Depression.

### Exposure assessments

Information on infant sex, birth weight to the nearest 50 grams (g), and birth length from crown to heel in centimeters (cm) was gathered from midwives' birth records in the National Archives of Iceland. Mean birth weight in Iceland is one of the highest in the world [23]. In the complete cohort, there was a low prevalence (2.9%) of infants weighing below 2.5 kilograms (kg). Therefore, lower birth weight was defined as infants weighing less than 3.0 kg (prevalence 4.9%). Ponderal index was calculated as birth weight in g/birth length in cm^3^×100. Yearly growth measures from ages 8 to 13 years including weight, height, and date of measurement were recorded by a school nurse and gathered from school health records.

Data on childhood growth was available for a total of 1500 individuals, representing 55% of participants in the current study. Despite this attrition, previous analysis has shown that anthropometric measures at birth and adult age do not differ markedly between subjects with and without growth data [24]. Furthermore, the mean height of subjects who had information on childhood growth available was compared to reference data for all public schools in Reykjavik [25]. At age 10 years, the average height difference from the reference values was 0.4 cm (men) and 0.1 cm (women) (data not shown) suggesting our growth data is a fair representation of school children in Iceland during this time. To determine differences in childhood growth patterns based on prenatal exposure to the Depression, mean BMI values from ages 8 to 13 for each birth year group were plotted against the current World Health Organization (WHO) BMI-for-age standards [26] for males and females separately.

### Outcome assessment

The longitudinal Reykjavik Study collected information on health and anthropometric measures at adult age. Body weight was measured to the nearest 100 g without shoes and height was measured with accuracy of ±0.5 cm. Subcutaneous skinfolds were measured with calibrated calipers to the nearest 1.0 mm [22]. Obesity was defined as body mass index (BMI) ≥30 kg/m^2^. Fasting blood glucose, serum triglycerides, and total cholesterol were measured after an overnight fast [27]. Impaired fasting glucose [28] was classified as fasting glucose ≥6.1 mmol/L and/or diagnosis or confirmation of type 2 diabetes. Dyslipidemia was defined as triglycerides >1.7 mmol/L and total cholesterol >6.2 mmol/L.

### Statistical analyses

The mean and standard error of the mean for birth weight and ponderal index were calculated separately by sex and yearly from 1925 to 1934. The mean and standard deviation (SD) or percentages were used to describe the cohort.

Linear regression analysis was performed for each outcome variable with birth year exposure to the Depression being a predictor and separately by sex. Maternal age and parity were included in all linear regression models as independent covariates. Participant age at examination was also included as a covariate when analyzing outcome variables collected in adulthood.

Logistic regression was used to estimate odds ratio (OR) of obesity, impaired fasting glucose, and dyslipidemia with the same covariates used in the linear regression models. Outcomes were adjusted for sex (when appropriate) however, women and men were also analyzed separately. Analyses included in the tables were not adjusted for participant's current or previous smoking habits as it post-dates birth year, however, data on percentage of current and previous smokers was obtained and is reported for descriptive purposes. Further modeling was performed to test for a recruitment effect by narrowing of study recruitment years and age range of participants, as well as addition of smoking variables although this data is not presented. All statistical analyses were carried out using SPSS 20.0 (IBM, New York). Significance was defined as *P*<0.05, 2 sided.

## Results

**Figure 1:**
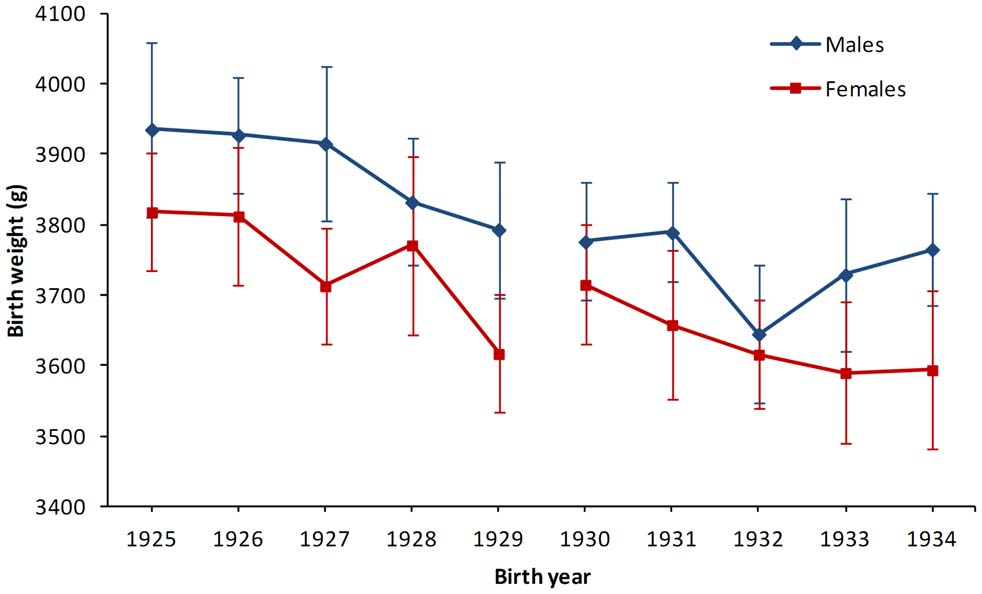
Yearly birth weight for men (diamond) and women (square) born between 1925 and 1934. Footer: Data are shown as means and the bars denote the standard error of the means. Birth weight is further stratified by sex and grouped by birth during pre-Depression (1925–1929) and during the Depression (1930–1934).

**Figure 2:**
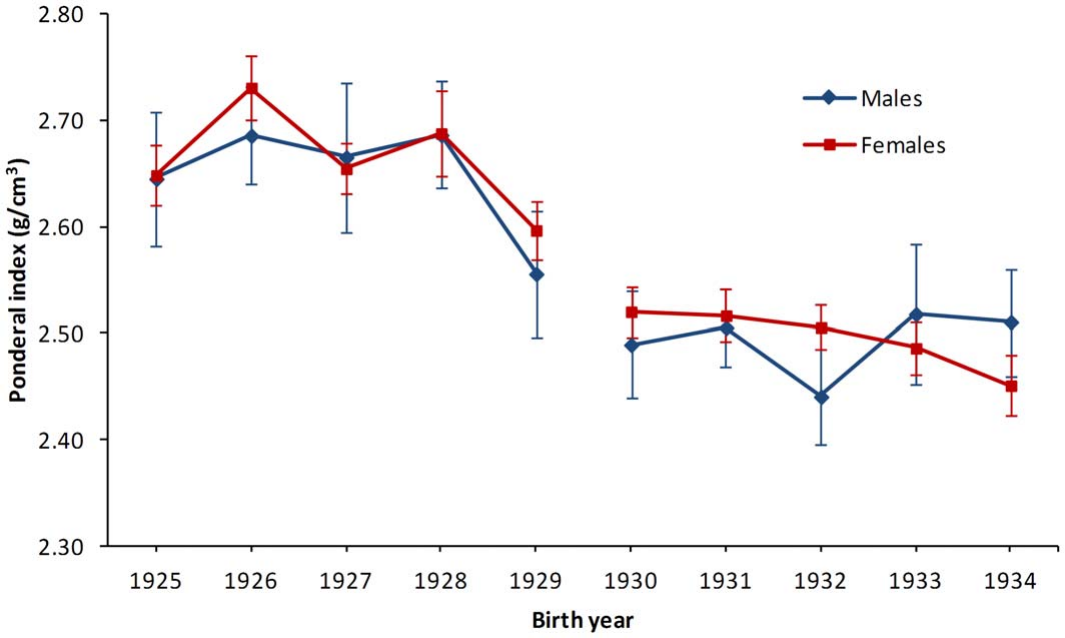
Yearly ponderal index^a^ for men (diamond) and women (square) born between 1925 and 1934. Footer: Data are shown as means and the bars denote the standard error of the means. Ponderal index is further stratified by sex and grouped by birth during pre-Depression (1925–1929) and during the Depression (1930–1934). ^a^ Ponderal index = (birth weight in g/birth length in cm^3^*100).

Figure 1 shows the mean birth weight by year of birth stratified by sex and Table 1, Panel A summarizes the birth characteristics. From birth year group born pre-Depression (1925–1929) to the group born during the Great Depression (1930–1934), there was a mean adjusted decrease in birth weight of 97 g for men and 70 g for women. Concurrently, ponderal index (Figure 2) decreased from 2.66 for males and 2.65 for females to 2.50 g/cm^3^ for both sexes.

**Table 1:** Characteristics at Birth, Age 10 Years, and at Study Recruitment, Mean (SD) or Percentage, for Men and Women Born Pre- or During the Great Depression.

	Men				Women			
	Pre-Depression	Depression	Δ	95% CI	Pre-Depression	Depression	Δ	95% CI
*Panel A: Birth characteristics* *	N = 685	N = 738			N = 687	N = 640		
Birth weight (g)	3880 (593)	3751 (531)	−97	−156, −39	3732 (555)	3638 (544)	−70	−129, −11
Ponderal index^a^ (g/cm^3^)	2.66 (0.34)	2.50 (0.30)	−0.15	−0.18, −0.12	2.65 (0.34)	2.50 (0.27)	−0.15	−0.18, −0.11
BW<3.0kg (%)	3.9	4.6	0.7	−	4.4	7.0	2.6	−
Ponderal index <2.6 g/cm^3^ (%)	45.7	66.9	21.2	−	42.4	64.3	21.9	−
*Panel B: Growth characteristics at age 10* *	N = 390	N = 401			N = 392	N = 317		
Height (cm)	137.7 (5.2)	140.0 (5.9)	1.7	1.0, 2.5	137.1 (5.2)	139.1 (5.9)	1.8	1.0, 2.6
Weight (kg)	31.2 (3.8)	32.3 (4.6)	1.0	0.4, 1.5	31.2 (4.4)	32.7 (5.2)	1.4	0.7, 2.1
BMI (kg/m^2^)	16.4 (1.3)	16.5 (1.5)	0.1	−0.1, 0.3	16.6 (1.6)	16.9 (1.9)	0.3	0.02, 0.5
								
*Panel C: Characteristics at study recruitment*	N = 685	N = 738			N = 687	N = 640		
Age	51.5 (4.6)	46.5 (5.1)	−5.0	−	52.3 (5.3)	51.0 (5.1)	−1.3	−
Current smoker (%)	57.1	62.0	4.9	−	41.9	44.6	2.7	−
Previous smoker (%)	21.2	21.6	0.4	−	14.0	20.1	6.1	−
Impaired fasting glucose (%)	4.9	2.5	−2.4	−	2.2	2.8	0.6	−
Dyslipidemia (%)	14.0	12.1	−1.9	−	6.8	7.7	0.9	−
Type 2 diabetes (%)	4.8	2.4	−2.4	−	2.0	2.5	0.5	−
Obese (% BMI ≥30 kg/m^2^)	11.9	13.6	1.7	−	10.3	13.0	2.7	−
								
*Anthropometrics* †								
Height (cm)	178.3 (6.0)	179.6 (6.3)	0.6	−0.2, 1.3	164.8 (5.1)	165.6 (5.4)	0.5	−0.04, 1.1
Weight (kg)	83.1 (12.3)	84.2 (13.0)	1.2	−0.3, 2.7	68.0 (11.2)	70.0 (11.6)	2.2	0.9, 3.4
BMI (kg/m^2^)	26.1 (3.5)	26.1 (3.6)	0.2	−0.2, 0.7	25.0 (3.9)	25.5 (4.1)	0.6	0.2, 1.1
Triceps skinfold (mm)	10.7 (7.2)	11.8 (8.0)	−0.2	−1.1, 0.7	20.6 (10.1)	22.2 (10.1)	1.9	0.8, 3.0
Subscapular skinfold (mm)	16.8 (7.6)	17.1 (8.0)	1.3	0.4, 2.2	18.8 (10.0)	21.5 (10.8)	3.3	2.2, 4.4
								
*Biomarkers* †								
Total cholesterol (mmol/L)	6.3 (1.0)	6.2 (1.0)	−0.16	−0.3, −0.04	6.4 (1.1)	6.2 (1.1)	−0.15	−0.3, −0.04
Triglycerides (mmol/L)	1.4 (0.9)	1.5 (0.8)	0.1	0.0, 0.2	1.1 (0.5)	1.1 (0.5)	0.06	0.0, 0.1
Fasting glucose (mmol/L)	4.6 (0.9)	4.5 (0.7)	−0.06	−0.2, 0.03	4.3 (0.6)	4.5 (0.9)	0.16	0.07, 0.23
								

Adjusted differences (Δ) and 95% Confidence Intervals are presented.

* Birth and growth characteristics at age 10 years adjusted for maternal age and parity.

† Adult anthropometrics and biomarkers adjusted for maternal age, maternal parity, and participant age at recruitment.

BW: birth weight; BMI: body mass index

a Ponderal index BW(g)/BL(cm)^3^×100.

Lower birth weight (<3.0 kg) was more common in infants with birth year exposure to the Depression compared to unexposed participants (Table 1, Panel A). Although differences were non-significant prevalence of lower birth weight rose from 3.9% to 4.6% and 4.4% to 7% for men and women, respectively. Among Depression-born infants there was a marked increase in ponderal index below 2.60 g/cm^3^, a cut-off used for describing thinness in infants [9], [29].

Childhood growth characteristics (Table 1, Panel B) show that at 10 years of age both boys and girls with birth year exposure to the Depression were greater in weight and height than their unexposed counterparts. However, only Depression-born girls had significantly higher BMI compared to girls born before the Depression. At study recruitment (Table 1, Panel C), women with birth year exposure to the Depression had higher mean BMI (adjusted Δ0.6 kg/m^2^, 95% CI 0.2, 1.1) and fasting blood glucose (adjusted Δ0.16 mmol/L, 95% CI 0.07, 0.23) compared to women born pre-Depression. Triceps and subscapular skinfold measures were also significantly higher among Depression-born women. Men born during the Depression had greater subscapular skinfold measures, yet, differences in BMI among men were negligible. Total cholesterol levels were slightly decreased among Depression-born men and women while triglycerides levels were slightly higher.

**Figure 3:**
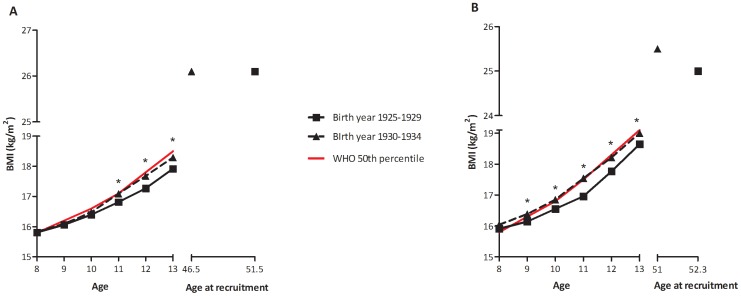
Mean BMI from 8–13 years by birth year group for A) men and B) women. Footer: Subjects born pre-Depression (1925–1929) were growing up during the Depression; while subjects born during the Depression (1930–1934) were growing up after the Depression and had faster BMI gain, growing closer to the 50^th^ percentile for the WHO BMI-for-age standards compared to their counterparts born earlier. *Significant difference between BMI values of participants born pre-Depression vs. during the Depression, adjusted for maternal age and parity (P<0.05).

Mean BMI from ages 8 to 13 stratified by birth year exposure to the Depression are shown in Figure 3A for males and 3B for females. When plotted against the WHO BMI-for-age standards, mean BMI from ages 8 to 13 of children born before the Depression followed approximately along the 25^th^ percentile while the BMI of Depression-born children was closer to the 50^th^ percentile. Growth differences based on birth year exposure were more pronounced among girls from ages 9 to 13 than among boys.

In Table 2, adjusted odds ratios for obesity, impaired fasting glucose, and dyslipidemia are presented with comparisons between birth year exposure to the Depression. In multivariate analysis, the combined OR for obesity was 1.40 (95% CI 1.09, 1.77). In separate analysis by sex, the odds of obesity remained significant only among women OR 1.43 (95% CI 1.01, 2.02) exposed prenatally to the Depression. Comparison of odds of impaired fasting glucose was not significant among women or among men. There was no marked difference in corresponding OR for dyslipidemia for either sex based on birth year exposure to the Depression. Addition of current smoking and previous smoking as categorical variables to the regression models did not change the results (data not shown).

**Table 2:** Adjusted Odds Ratios for Obesity, Impaired Fasting Glucose, and Dyslipidemia at Study Recruitment in Adulthood Comparing Men and Women Born During the Great Depression to Pre-Depression.

	All^a^		Males^b^		Females^b^	
Variable	OR	95% CI	OR	95% CI	OR	95% CI
Obesity (BMI ≥30 kg/m^2^)	1.40	1.09, 1.77	1.27	0.89, 1.81	1.43	1.01, 2.02
Impaired fasting glucose^c^	0.98	0.62, 1.57	0.75	0.40, 1.41	1.41	0.69, 2.90
Dyslipidemia^d^	0.95	0.73, 1.24	0.72	0.51, 1.03	1.22	0.80, 1.86

Reference group: participants born Pre-Depression (1925–1929).

BMI: body mass index; OR: odds ratio, CI: confidence interval

a. Adjusted for maternal age, maternal parity, participant age at examination and sex.

b. Adjusted for maternal age, maternal parity, and participant age at examination.

c. Impaired fasting glucose: fasting glucose ≥6.1mmol/L and/or diagnosis or confirmation of type 2 diabetes.

d. Dyslipidemia: total cholesterol >6.2 mmol/L and triglycerides >1.7 mmol/L.

In order to further examine whether participant age influenced outcomes due to a recruitment effect we narrowed the age range of participants from 33–65 years to 45–55 years as well as recruitment year from 1967–1991 to 1974–1983. Adjusted combined OR for obesity remained significant 1.59 (95% CI 1.12, 2.26), with a stronger association among women 2.16 (95% CI 1.25, 3.76) while the combined odds ratios for impaired fasting glucose and dyslipidemia remained non-significant (data not shown).

## Discussion

In this Icelandic cohort we investigated the impact of the Great Depression on size at birth and adult obesity, impaired fasting glucose, and dyslipidemia in 2750 Icelanders living in Reykjavik. We found that infants with birth year exposure to the Depression were of lower birth weight. In adulthood, Depression-born females had a higher mean BMI and greater odds of being obese but this effect was not observed in males.

With regards to size at birth, there was a significant decline in birth weight and ponderal index from 1925 to 1934 indicative of infants being born thinner as the economic impact of Depression progressed. Although birth weight still remained relatively high, the decreasing ponderal index is suggestive of increasing fetal undernutrition along with economic and environmental influences as there is a distinct and consistent drop after the initial onset of the Depression in Iceland in 1930.

The 1920s and 1930s in Iceland were characterized by increased migration into Reykjavik and changes in birth weight may also be a consequence of overcrowding which can negatively affect birth size [30]. The current cohort includes only city residents and may capture some of this early migratory trend into Reykjavik. The flux in birth weight may be reflective of the shifting urban population however, as all participants were born and living in Reykjavik when recruited into the study, it is unlikely that this had a substantial influence on the outcomes measured.

In addition to birth size, studies have suggested accelerated postnatal growth in thin infants may contribute to later disease [31]. Between the ages 8 to 13 years, both Depression-born boys and girls were taller and heavier than their counterparts born prior to the Depression, despite having a similar BMI at age 8. As the children grew older, those with birth year exposure to the Depression had faster BMI gain which may have placed them at greater risk of developing obesity in adulthood.

It should be noted that the environment during which these participants were growing was quite different. Children born between 1925 and 1929 were growing-up during the Depression where access to food was more difficult and costly [17], while those born during the Depression (1930–1934) were on average growing in a more plentiful environment. Historically, this was primarily due to improvements in the fortunes of the Icelandic economy during the Second World War. Iceland experienced higher economic growth than most European countries from increased exports and greater demand created by the British and later American occupation forces in the country. The simultaneous economic change was likely linked with increased efforts by parents of Depression-born children to encourage weight gain. The relative contribution of an adverse prenatal environment during the Depression coupled with an improved situation may have contributed to the differences in childhood growth patterns.

In adulthood, we observed higher fasting glucose levels and increased obesity only among women with birth year exposure to the Depression. We observed virtually no difference in BMI or odds of obesity among men. There is evidence that increasing glucose intolerance and obesity can coincide with transition to an environment of better nutrition [32]. However, both sexes experienced comparable environmental exposures suggesting the gender differences did not arise from the same etiologies. Previous analysis in this cohort also found sex-specific differences, with stronger inverse associations between birth weight and truncal fat [22] and glucose intolerance [19] among women at adult age. Similar findings were published on the Hertfordshire cohort, where fasting glucose measures were more strongly inversely associated with birth weight in women [33].

We did not find the odds of impaired fasting glucose or dyslipidemia for either sex to be linked to birth year exposure to the Depression. The relatively high birth weight in Icelandic infants has been proposed to have a protective effect with respect to diseases (i.e. coronary heart disease, hypertension) related to small size at birth [19]. In several epidemiological studies higher birth weight was associated with increased lean body mass but not adiposity later in life [34]–[36]. The difference in birth weight between infants born prior to versus during the Depression may reflect a lower proportion of lean body mass in the Depression-born infants. As muscle is an important site for glucose homeostasis, this could lead to abnormalities in blood glucose [37] which may explain the slightly higher fasting glucose levels we observed in adult women.

While the findings of greater cardiometabolic risk factors primarily among women corroborate those from other epidemiological cohorts [33], [38], there are several possible limitations. Data on maternal birth or adult size, gestational age or placental function was not available for analysis and their effect on birth size in this cohort is unknown. However, adjustments for strong predictors of birth weight (i.e. maternal age and parity) performed in this current study did not affect the outcomes.

Although the participants were recruited at various ages (33–65 years), our findings do not appear to be influenced by a recruitment effect. In addition, Vilbergsson et al [26] previously reported that prevalence of non-insulin dependent diabetes in this cohort was consistent between the recruitment years 1967–1991. Thus, with regards to impaired fasting glucose this allows for a fair comparison between the subjects based on calendar year exposure to the Great Depression. The relationship between birth size and adult hypertension has been previously discussed in this cohort [24]. We observed minor decreases in systolic and diastolic blood pressure which coincided with increasing use of anti-hypertensive medication for those recruited later in the cohort. Adjusting for this trend would not be possible without adjusting for calendar year therefore blood pressure was not included in this current analysis.

This cohort represents a population that was undergoing both economic and environmental changes during development in utero and early childhood [16]. The individual impact of the Depression cannot be fully quantified, yet on a cohort level the distinct increase in infants with low ponderal index suggests the environmental strain was considerable enough to affect maternal health condition and birth size of offspring. Ultimate height and weight is a result of the interaction between many factors, but it appears that even restricted but non-famine like conditions experienced in utero (based on birth year) can modestly influence risk of later obesity while other cardiometablic risk factors (i.e. triglycerides, cholesterol, and glucose levels) seem to be less sensitive to these conditions. We also found that women were more susceptible to these conditions, which is in line with previous findings from this cohort.

## Conclusion

In this current paper, we have presented that birth year exposure to the Great Depression was associated with offspring who were lighter at birth and Depression-born women had a greater likelihood of being obese in adulthood. With the increasing frequency of obesity-related diseases, the ability to identify environmental triggers that may negatively impact birth size in offspring and continue to affect weight status in later life is becoming critical. Pregnancy during periods of economic downfalls may inadvertently stress the fetus initiating a pathway favoring obesity development. Our findings contribute to the existing literature on environmental influences on size at birth and disease in middle-aged adults and emphasize the importance of accounting for both prenatal and postnatal environmental influences when exploring these associations.
